# Propofol Sedation by Pediatric Gastroenterologists for Endoscopic Procedures: A Retrospective Analysis

**DOI:** 10.3389/fped.2019.00098

**Published:** 2019-03-26

**Authors:** Aya Khalila, Itai Shavit, Ron Shaoul

**Affiliations:** ^1^Pediatric Gastroenterology and Nutrition Institute, Ruth Children's Hospital, Rambam Health Care Campus, Haifa, Israel; ^2^Faculty of Medicine, Technion Israel Institute of Technology, Haifa, Israel; ^3^Pediatric Emergency Department, Ruth Children's Hospital, Rambam Health Care Campus, Haifa, Israel

**Keywords:** sedation, endoscopies, children, safety, non-anesthesiologist administered propofol

## Abstract

**Background:** There is a substantial literature on the favorable outcome of propofol administration by non-anesthesiologists for endoscopy in adults; however, very few data are currently available on propofol sedation by pediatric gastroenterologists. Aims: to evaluate the safety of propofol sedation by pediatric gastroenterologists.

**Methods:** A retrospective chart review of all children who were sedated by pediatric gastroenterologists in three Northern Israeli hospitals over a 4 years period Demographic and medical characteristics and any data regarding the procedure were extracted from patient's records. The main outcome measurements were procedure completion and reported adverse events.

**Results:** Overall, 1,214 endoscopic procedures for were performed during this period. Complete data was available for 1,190 procedures. All children sedated by pediatric gastroenterologists were classified as ASA I or II. Propofol dosage (in mg/kg) inversely correlated with patient age. The younger the child the higher the dose needed to reach a satisfactory level of sedation (*r* = −0.397, *p* < 0.001). The addition of fentanyl significantly decreased propofol dosage needed to provide optimal sedation, *p* < 0.001. Nine (0.7%) reversible adverse events were reported. All the procedures were successfully completed and all patients were discharged home.

**Conclusions:** We conclude that our approach is safe in children as it is in adults and can be implemented for children with ASA I, II.

## Introduction

The amount of gastrointestinal endoscopies (GE) performed in childhood has significantly increased over the last two decades, improving both diagnosis, and treatment of pediatric gastrointestinal diseases (1). This has also increased the demand for safe and effective procedural sedation. Pediatric gastrointestinal procedures, such as esophagogastroduodenoscopy (EGD) and colonoscopy require substantial immobilization for successful performance and are uncomfortable and emotionally disturbing for children (2). Some gastroenterologists use light intravenous sedation, typically benzodiazepines, and opioids titrated to levels consistent with conscious sedation (2–4). Most centers these days regard light sedation as inadequate, and regularly use general anesthesia instead to perform these procedures (2–4). Nevertheless, general anesthesia is only available in a limited number of centers because of shortness of anesthesiologists.

Propofol (2,6-diisopropyl-phenol) is an ultra-short acting sedative agent. It is a phenolic derivative with satisfactory sedative, hypnotic, antiemetic, and amnesic properties. Propofol is highly lipophilic and thus can rapidly cross the blood-brain barrier, resulting in an early onset of action. Regardless of the depth or length of the sedation period, propofol has a short recovery profile (5), there is a conspicuous literature on the favorable outcome of propofol administration by non-anesthesiologists for endoscopy in adults, including a meta-analysis of randomized controlled trials revealing that its use is favored due to rapid onset and offset of action, fast recovery time, and high patient and physician satisfaction (3–17). However, there is no available data on propofol sedation for pediatric gastrointestinal endoscopies by pediatric gastroenterologists.

In the Haifa region of Israel, gastroenterological endoscopies are mainly performed in three medical centers; Rambam Health Care Campus (RHCC), Elisha Medical Center (EMC), and Assuta Medical Center (AMC). In these centers, pediatric gastrointestinal endoscopies are performed by qualified pediatric gastroenterologists, highly skilled and specially trained in pediatric sedation that have been using propofol since 2008 (18). In RHCC, sedations for gastrointestinal endoscopies are performed by a heterogenic group of staff pediatric gastroenterologists and fellows in pediatric gastroenterology. In AMC and EMC sedations are performed by a single qualified pediatric gastroenterologist.

We aimed to retrospectively evaluate the safety and effectiveness of propofol sedation by pediatric gastroenterologists for gastroenterological endoscopies.

## Methods

### Study Design

We performed a retrospective chart review of all children who were sedated by pediatric gastroenterologists in RHCC, EMC, or AMC between 1.1.2008 and 31.12.2011.

Demographic and medical characteristics and any data regarding the procedure process were extracted from the electronic records. Data collected included gender, age and weight, type of procedure, medical center (RHCC, AMC, or EMC), sedation medications, and dosages, type of procedure, and any serious adverse events during sedation (SAEDS). Sedation protocol defines SAEDS as: “death, cardiac arrest, endotracheal intubation, hospitalization due to an adverse event, hypoxia (saturation ≤ 90%), apnea (discontinuation of breathing), aspiration (coughing or chocking associated with observed gastric contents in the mouth), and laryngospasm (upper airway obstruction with oxygen desaturation caused by closure of the vocal cords), and hypotension (blood pressure below two standard deviations (SDs) of the mean for age and gender) requiring treatment with volume replacement (6).”

Propofol was manually titrated to the desired level of sedation.

The protocol for the research project was approved by a suitably constituted Ethics Committee of RHCC within which the work was undertaken. All human studies have been reviewed by the appropriate ethics committee and have therefore been performed in accordance with the ethical standards laid down in an appropriate version of the Declaration of Helsinki (as revised in Brazil 2013), available at http://www.wma.net/en/30publications/10policies/b3/index.html.

### Sedation Protocol

Based on Israeli MOH pediatric sedation guidelines, in RHCC, EMC, and AMC all children undergoing gastrointestinal endoscopies are sedated by a pediatric gastroenterologist if the child is older than 24 months, has an American Society of Anesthesiologists (ASA) physical status classification of ≤ 2, and has been fasting for ≥6 h. All children under 2 years of age and with ASA score above 2 had been sedated by an anesthetist. Our protocol recommends using propofol with midazolam in separate doses, with midazolam given first to decrease anxiety. All children in the study received 1 mg of midazolam irrespective of weight (all were above 10 kg). The pediatric gastroenterologist and a nurse trained in pediatric sedation were both responsible for patient monitoring. Monitoring includes verifying proper head and neck position and airway patency throughout the procedure, heart rate, and oxygen saturation monitoring. Oxygen supplementation was routinely provided during the procedure. The sedation target was immobilized patient and that the endoscope will be easily inserted. We start with a fixed dose of 1 mg midazolam (serves as anxiolytic drug). It is followed by a slow bolus of fentanyl for colonoscopy in all institutions and for all endoscopic procedures in EMC and AMC). This is followed by 1.0 mg/kg of propofol. We monitor heart rate, oxygen saturation and level of sedation and add boluses of 0.5 mg/kg as needed to achieve and maintain appropriate sedation.

In RHCC, another pediatric gastroenterologist, experienced in sedation is responsible for delivering the sedation. In AMC and EMC the drug is delivered similarly to adult procedures by the endoscopist or by the sedation experienced procedure nurse (third hand).

Patients were discharged from the hospital after at least an hour and after complete recovery, defined by the presence of normal vital signs with the patient being fully awake, without any complains of nausea or vomiting. No known post discharge adverse events were noted.

Written informed consent was signed by the children's caregivers prior to each procedure.

### Statistical Analysis

Statistical analysis was performed using SPSS version 21. Quantitative parameters were presented by using means and SDs, and categorical parameters were presented by frequencies and percentage. One way Anova with *post-hoc* tests and *t*-test were used for differences between quantitative parameters in different groups (type of procedure, gender, hospital etc.). Linear correlations between quantitative parameters were used by Pearson correlation. Differences between categorical parameters were used by Pearson chi-square. Linear regression for prediction propofol per weight was used with three independent parameters (type of procedure, gender, type of hospital) *P* < 0.05 was consider as significant.

## Results

Overall, 1,214 diagnostic endoscopic procedures have been performed during this period. Four hundred and fifty-five (37.5%), 365 (30%), and 394 (32.5%) endoscopies were performed in RHHC, AMC and EMC, respectively. Complete data was available for 1,190 procedures. Nine hundred and seventy one (80%) were upper endoscopies, 123 (10.1%) were colonoscopies and 119 (9.9%) were combined upper-and-lower endoscopies. Seventy one sedations have been carried out by an anesthetist. Demographic and medical characteristics are shown in Table 1.

**Table 1:** Patients' characteristics.

	**RHCC (*n* = 454)**	**AMC+EMC (*n* = 759)**	***P*-value**
**DEMOGRAPHIC CHARACTERISTICS**			
Age, mean (SD), y	8.7 (5.3)	13.5 (2.6)	0.001
Weight (%), kg	32.25 (19.2)	48.9 (15.2)	0.001
Male (%)	208 (45.7)	365 (48.1)	0.44
**ENDOSCOPY**			
Upper GI, *n* (%)	353 (77.5)	618 (81.5)	0.1
Lower GI, *n* (%)	43 (9.5)	80 (10.6)	0.55
Upper and lower GI, *n* (%)	60 (7.9)	59 (13)60 (7.9)	0.005
Adverse events, *n* (%)	9 (2)	0 (0)	–

*SD, Standard Deviation; GI, Gastrointestinal; RHCC, Rambam Health Care Campus; AMC, Assuta Medical Center, EMC, Elisha Medical Center*.

### Propofol Dosage

Mean propofol dosage is reported in Table 2. Propofol dosage was significantly higher in RHCC compare to AMC and EMC (4.06 vs. 2.28 mg/kg, *p* < 0.001).

**Table 2:** Propofol dosage (mg/kg) in the different settings.

**Propofol dosage (mg)/weight (kg)**			
	***N***	**Mean ± SD**	***P*****-value**
**GENDER**			
Male	566	3.082 ± 1.67	*P* = 0.006
Female	624	2.818 ± 1.618	
Total	1,190	2.944 ± 1.65	
**HOSPITAL**			
RHCC	444	4.056 ± 1.94	*P* < 0.001
AMC + EMC	746	2.282 ± 0.96	
Total	1,190	2.944 ± 1.65	
**ENDOSCOPY TYPE**			
Upper	951	2.683 ± 1.39	^a^*p* < 0.001
Lower	120	3.595 ± 2.11	^b^*p* < 0.001
Upper/lower	118	4.398 ± 2.08	^c^*p* < 0.001
Total	1,189	2.944 ± 1.65	
Anesthesiologist	71	5.69 ± 2.78	*p* < 0.001
Gastroenterologist	1,119	2.77 ± 1.39	
**COMPLICATIONS**			
No	1,181	2.928 ± 1.64	*p* < 0.001
Yes	9	4.986 ± 1.68	

a *Significant differences between EMC hospital vs. RHCC, hospital*.

b *Significant differences between EMC hospital vs. AMC, hospital*.

c *Significant differences between RHCC hospital vs. AMC, hospital*.

**Figure 1:**
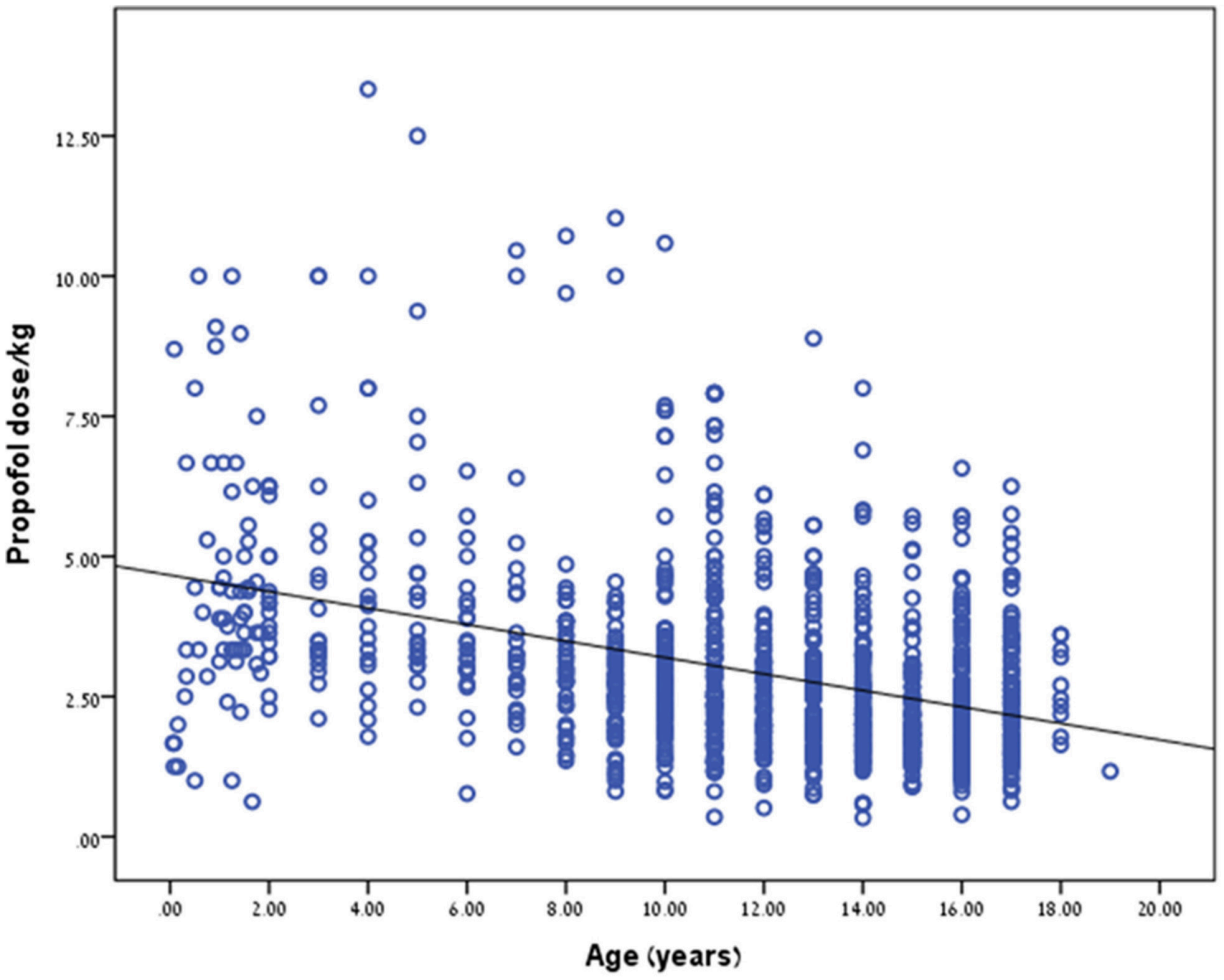
Relation between patient age and propofol dose (mg/kg).

Propofol dosage (in mg/kg) correlated with patient age. The younger the child the higher the dose needed to reach a satisfactory level of sedation (*r* = −0.397, *p* < 0.001, Table 2; Figure 1). We did further analysis according to the procedure type and the findings remained the same. *r* = −0.588, *p* < 0.0001 for colonoscopy, *r* = −0.487, *p* < 0.0001 for upper endoscopy and *r* = −0.634, *p* < 0.0001 for combined procedures. Interestingly, females required a less dosage of propofol than boys in order to reach an appropriate level of sedation, *p* = 0.006.

Propofol dosage was significantly higher in combined upper-and-lower endoscopy compare to upper endoscopy and lower endoscopy (*p* < 0.001, for all comparisons, Table 2).

**Figure 2:**
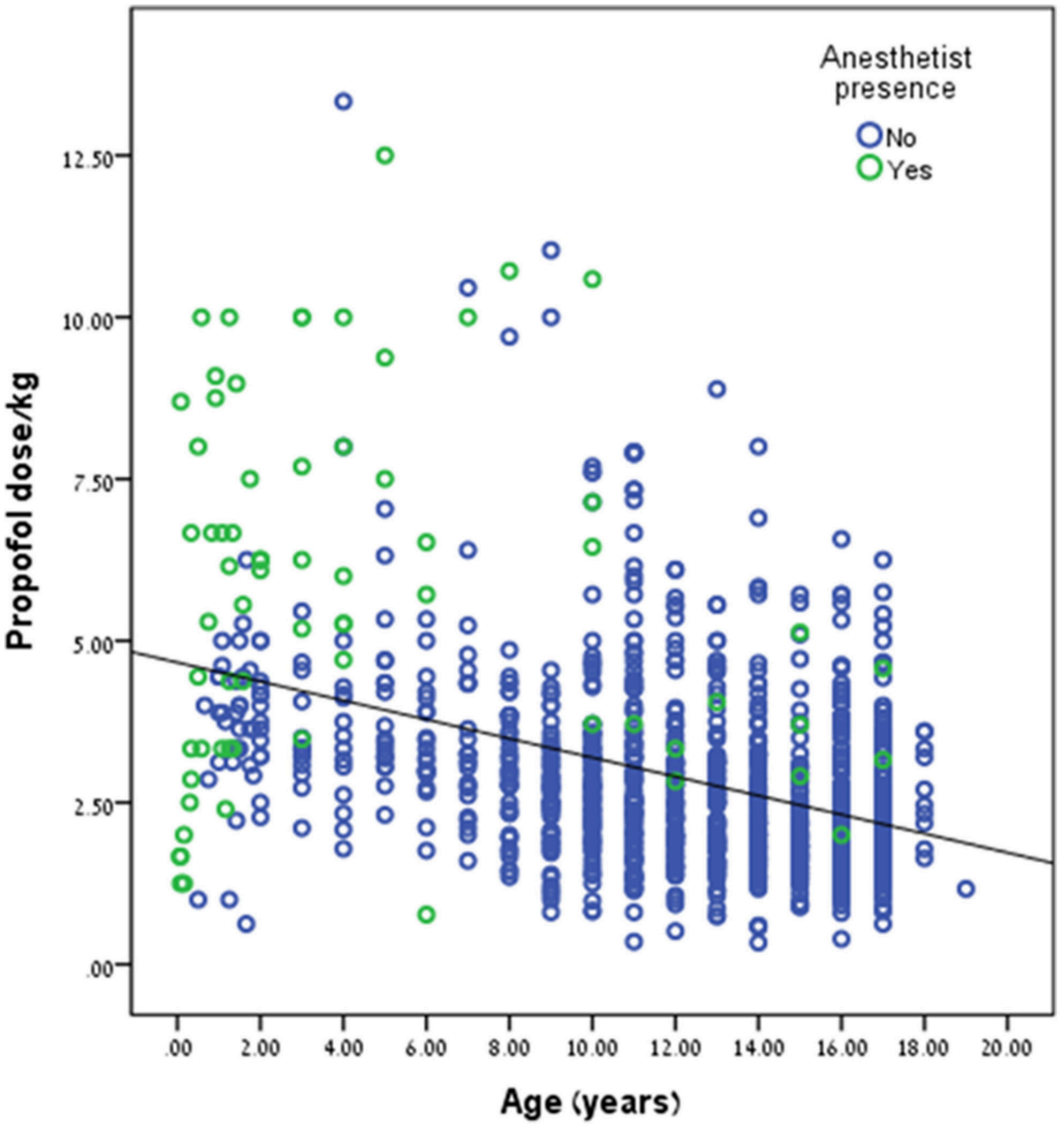
Relation between presence of anesthetist and propofol dose.

**Figure 3:**
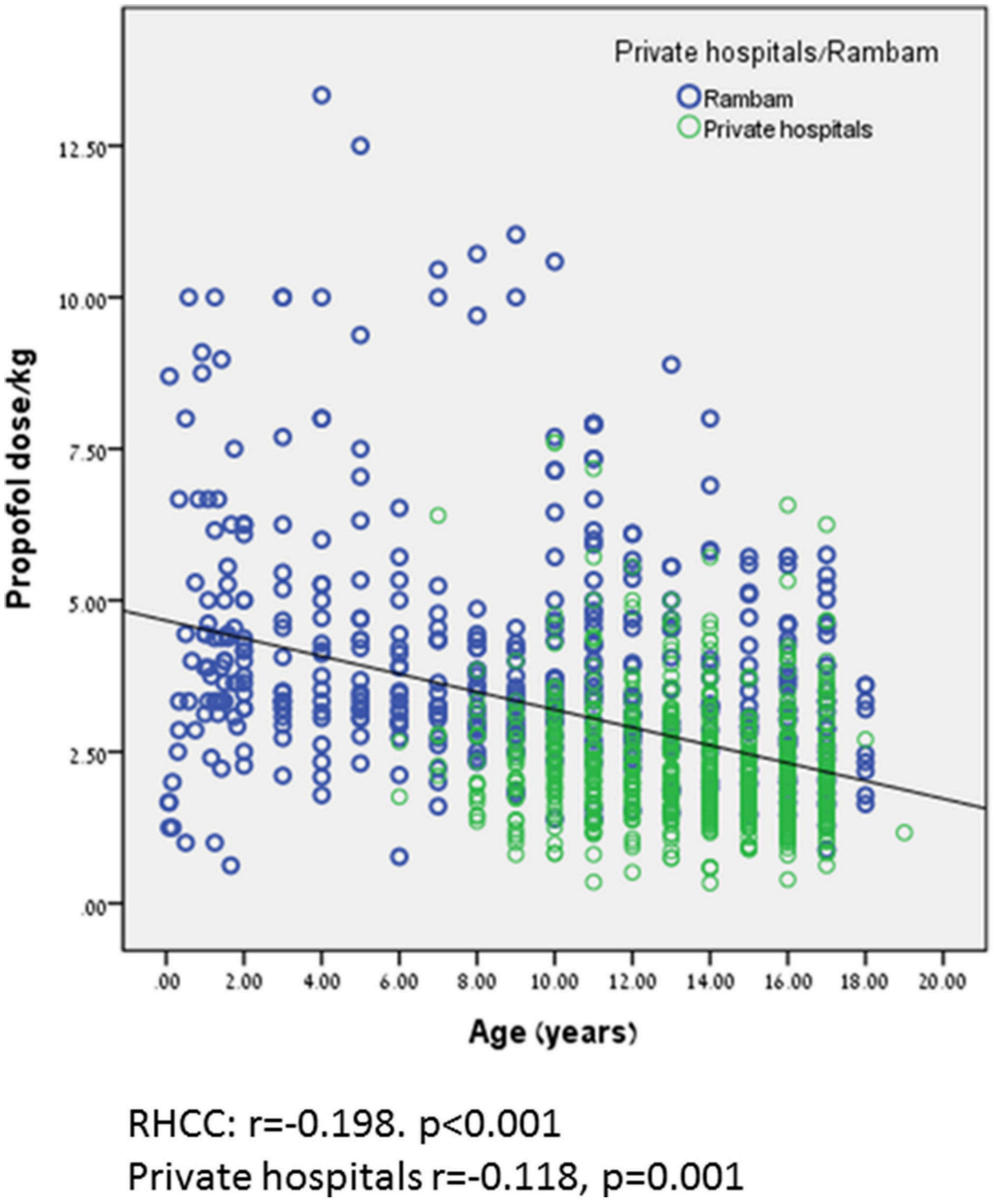
Relation between procedure location and propofol dose/kg.

Propofol dose/kg was significantly higher during anesthetist presence compared to pediatric gastroenterologist, 5.69 ± 2.78 kg vs. 2.77 ± 1.39 mg/kg, *p* < 0.001, respectively. This trend can be seen in Figure 2. Propofol dose/kg was also significantly higher in RHCC hospital compared to the other hospitals (Figure 3).

The mean midazolam dose was 0.0385 ± 0.03220 and the mean fentanyl dose (864 patients) was 0.001434 ± 0.0006189 mg/kg.

### SAEDS

Nine (0.7%) SAEDS were reported, all in RHCC. No deaths were reported, no patient was reported to be admitted due to an adverse reaction, and no patient required placement of endotracheal tube. All the procedures were successfully completed and all patients were discharged home. No significant hypotension, or cardiac arrhythmias were reported. In one case, an 8 years old developed laryngospasm with oxygen desaturation during an upper endoscopy. The sedation for this procedure was given by an anesthetist. He was successfully treated with bag-mask ventilation and dexacort and was discharged asymptomatic after few hours of observation. Eight children (7 upper endoscopies and 1 lower endoscopy) experienced short episodes of oxygen desaturation that resolved with repositioning of the airway. Children who experienced SAEDS received higher doses of propofol 4.9 ± 1.68 vs. 2.92 ± 1.64 mg/kg, *p* < 0.001.

### The Effect of Fentanyl on Propofol Dose

In order to examine the impact of combined sedation with fentanyl, propofol, and midazolam on propofol dosage, we compared between all children who received combinative sedation of the three sedative agents and children who did not received fentanyl. Overall, 864 (852 for whom complete data was available) children received combinative sedation of propofol and fentanyl. The addition of fentanyl significantly decreased propofol dosage needed to provide optimal sedation, *p* < 0.001 (Table 2).

## Discussion

The primary goals of most sedation regimens for pediatric endoscopic procedures areto ensure relaxed and safe atmosphere for the patient throughout the procedure. Secondary and often desirable goals of sedation are to effect peri-procedural amnesia, maximize procedural efficiency, minimize recovery times, and maintain cost-effectiveness (4). Although general anesthesia (GA) is considered safe and effective in providing comfort and amnesia, GA requires expertise and has been viewed as not being cost effective for pediatric endoscopies (7). This together with lack of trained anesthesiologists pushes toward the practice of sedation by non-anesthesiologist providers during endoscopies. The use of IV sedation by pediatric endoscopists before the introduction of propofol was associated with a high risk for agitation, which adversely affect the quality of procedures for both patients and clinical staff (4). One way of lowering the incidence of patient agitation during pediatric endoscopies is to use propofol sedation, either as an isolated IV administration or in combination with other sedative drugs. Propofol has been shown in multiple studies to be highly effective at inducing sedation in children who are undergoing both upper and lower endoscopy, and provides excellent amnesia for the procedure (8–10). The introduction of propofol into adult practice and the data gained on its safety in pediatric procedures and pediatric emergency departments (11) has pushed toward its use in pediatric gastroenterology suites. This is the largest series of propofol administered by pediatric gastroenterologists ever reported to our knowledge.

Heuss et al. (12) reported that 43% of 180 Swiss endoscopists who replied to the survey use propofol without the assistance of an anesthesiologist regularly, mainly in a hospital setting. They had performed a total of 82,620 procedures. The morbidity in this group of patients was 0.19%, with no cases of mortality.

In a meta-analysis of 12 original studies including 1,161 adult patients of whom 634 received propofol, and 527 received midazolam, meperidine, and/or fentanyl. The pooled odds ratio with the use of propofol for developing hypoxia or hypotension for all the procedures combined was 0.74 (95% confidence interval [CI], 0.44–1.24); for EGD, 0.85 (95% CI, 0.33–2.17) and for colonoscopy, 0.4 (95% CI, 0.2–0.79) (13). A more recent meta-analysis (14) on 1,798 adult patients, of whom 912 received propofol only and 886 received traditional sedative agents supported these findings and concluded that propofol is safe and effective for gastrointestinal endoscopy procedures and is associated with shorter recovery and discharge periods, higher post-anesthesia recovery scores, better sedation, and greater patient cooperation than traditional sedation, without an increase in cardiopulmonary complications.

Based on that several position statements (15, 16) the safety profile of non-anesthesiologist administered propofol (NAAP) is equivalent to that of standard sedation with respect to the risks of hypoxemia, hypotension, and bradycardia for both upper endoscopy and colonoscopy. For EGD, colonoscopy, ERCP, and EUS, the time for sedation induction is shorter with NAAP than with standard sedation, recovery time for these procedures when using NAAP is shorter than for standard sedation with a narcotic and a benzodiazepine. Patient satisfaction with NAAP is equivalent or slightly superior to that with standard sedation.

Larsen et al. (17) reported the safety of propofol sedation by a pediatric intensivist for 4,716 pediatric outpatient procedures of which 2,332 (49%) were gastrointestinal. 56% were <10 years. For this group they had 355 minor complications (15.2%) (transient requirement of oxygen by nasal cannula or positive pressure ventilation by mask, airway repositioning by jaw thrust, or oropharyngeal suctioning to improve oxygen saturation) and one major complication (0.04%) at the 1–10 years age group in a child with glycogen storage disease and adenoid hypertrophy. They concluded that propofol sedation by a pediatric intensivist is a safe sedation technique in the pediatric outpatient setting. Barbi et al. (18) assessed the safety and efficacy of procedural sedation with propofol by non-anesthesiologist pediatric sedation unit using intravenous propofol. Transient desaturation resolving spontaneously occurred in 134 (12.7%) of 1,059 patients. Major desaturation requiring a short course of ventilation occurred in 4 (0.8%) of 483 patients undergoing upper endoscopies. The same group prospectively reported 3 years later (19) the use of propofol for 811 upper gastrointestinal endoscopy in children (ASA grades I–II), administered by specially trained pediatricians. None of the patients required intubation. Stridor with signs of upper airway obstruction occurred in 14 of the 811 procedures (1.7%). Major desaturation requiring a short course of ventilation occurred in six procedures (0.7%), and transient desaturation that resolved spontaneously occurred in 97 of the procedures (12%).

Van Beek et al. (20) recently reviewed 6 RCTs (*N* = 561 procedures) and 4 non-RCTs (*N* = 3,322 procedures) examining the safety and/or effectiveness of propofol based pediatric sedation. The majority of published propofol sedations (3,420/3,883; 88.1%) were performed by non-anesthesiologists [pediatric intensivists (8, 17) or specifically trained pediatricians (18, 19)]. They concluded that Propofol-based procedural sedation is safe. On a total of 3,883 reported propofol-based sedations, major respiratory complications like total airway obstruction, deep hypoxia, or apnea occurred 11 times (0.3%). They emphasize that mild respiratory events occur frequently and major complications may happen rarely, but adverse events do not occur more frequently compared with other sedation regimens. No cases of intubation, resuscitation, permanent sequelae, or death were reported. This is consistent with our findings.

Milius et al. (21) found that the mean dose of propofol required for female patients was 3.7 vs. 3.4 mg/kg for males (*p* = 0.3). In our study we observed the opposite trend (girls needed significantly less propofol dose/kg to achieve same sedation level). Another finding in their study was that the mean doses of propofol for patients ≤ 9 years, 10–12 years, and >12 years were 3.2, 3.9, and 3.9 mg/kg, respectively (*p* = 0.25). We noted an opposite trend in our study (higher doses/kg for younger patients). These opposite trends may be attributed to a much larger population in our study. The higher propofol dose/kg in procedures performed by anesthetist may be attributed to less experience in gastrointestinal sedation and dealing with younger and more complicated patients. The differences noted in propofol dose/kg between RHCC and the other hospitals are probably related to older age in AMC and EMC hospitals (although difference is still noted after correction for age), a single highly experienced endoscopist compared to mixed population of residents and young seniors performing the procedures in RHCC.

The combination of propofol with midazolam and fentanyl has been shown to decrease the amount of propofol and/or decrease recovery time in adults (22, 23). It has been shown to improve sedation quality in children. (10) We routinely add midazolam to our sedation and the addition of fentanyl has been shown to significantly reduce propofol dose in our study as well.

Since propofol has a narrow therapeutic range and there is no specific antagonist available, the administration of propofol had been restricted primarily to anesthesiologists and trained nurse anesthetists in order to manage airway in emergency (24). However, propofol has been noticed on account of the rapid time to onset and recovery time, in addition to the better or similar patient satisfaction (25, 26). Propofol has also been proven to reduce post-procedural hypoxemic events (27). Some recent studies suggest that propofol can be safely administered to children by non-anesthesiologists who specifically trained to follow established safety guideline (17–20, 28, 29).

The main limitations of the study are its retrospective nature. Nevertheless, all adverse events (minor and major) are well documented in patient's chart.

In their recently published review on sedation for gastrointestinal endoscopy in children by non-anesthesiologists, Orel et al. (30) denote that in many countries, including a majority of European countries and in parts of the United States, the limited availability of anesthesiology teams and limited organizational considerations represents a medical dilemma and an alternative should be sought. They provide evidence for sedation schemes, including propofol, which could be safely and efficiently performed by non-anesthesiologists as long as they are adapted to international, national and local legislation and institutional practice.

We practice as suggested by Bartkowska-Sniatkowska et al. that children with congenital defects and serious coexisting diseases (ASA ≥ III) must be managed by pediatric anesthesiologists (31). We conclude that our approach is safe in children as it is in adults and should be implemented for children with ASA I, II in countries suffering from anesthetists shortage.

## Author Contributions

AK contributed to this submitted work by reviewing patient charts and collecting data, and analyzing and interpreting the results. She gave final approval of the version to be published. IS contributed to this submitted work by assisting with sedation protocols, helped with writing of the manuscript. He gave final approval of the version to be published. RS contributed to this submitted work by designing the study and the variables to be investigated and by guiding the process. He wrote the manuscript.

### Conflict of Interest Statement

The authors declare that the research was conducted in the absence of any commercial or financial relationships that could be construed as a potential conflict of interest.
